# Grand Challenge in Plant Virology: Understanding the Impact of Plant Viruses in Model Plants, in Agricultural Crops, and in Complex Ecosystems

**DOI:** 10.3389/fmicb.2017.00860

**Published:** 2017-05-24

**Authors:** Hélène Sanfaçon

**Affiliations:** Agriculture and Agri-Food Canada, Summerland Research and Development CentreSummerland, BC, Canada

**Keywords:** plant virology, plant-virus interactions, antiviral defense, RNA silencing, virus resistance, virus genome, metagenomic analysis, viral proteins

The history of virology starts with a plant virus, tobacco mosaic virus, being identified as a novel type of infectious agent in 1898 (Creager et al., 1999). Since then, the ubiquity of viruses has become evident as well as their absolute dependence on their hosts. Common themes have emerged across all virology sub-disciplines, including the highly adaptable nature of viruses and their ability to harness and modify the host resources to complete their infection cycle. The last 120 years of plant virology research have also highlighted unique adaptations to the specific challenges posed with infecting plants. In this article, I will briefly discuss a few selected research areas that have transformed this dynamic field of research in recent years and I will also highlight challenges that lie ahead. This article is also meant as an invitation for plant virologists to join the discussion through the Virology Specialty platform. Indeed, Frontiers in Microbiology and Frontiers in Plant Sciences have recently joined forces to regroup all plant virology article submissions into the Virology Specialty, which is now cross-listed in both journals.

Inevitably, the first characterized plant viruses were those that cause serious diseases in cultivated crops. While some crops are amenable to molecular studies (e.g., members of the family *Solanaceae*), others are more difficult to work with. Luckily for virologists, many plant viruses have a wide host range and can infect well-characterized model plants, in particular *Arabidopsis thaliana*, with a genome sequence elucidated two decades ago and extensive genetic resources (Goodman et al., 1995) and *Nicotiana benthamiana*, a plant particularly susceptible to virus infection (Goodin et al., 2015). The common yeast has also emerged as a powerful model host for some plant viruses, allowing for identification of a large number of interacting host factors, many of which were later validated in plants (Nagy et al., 2014). Most studies have focussed on model plant viruses, generally selected for their high replication rate, the availability of infectious clones and their wide host range (Scholthof et al., 2011). As highlighted below, these simplified model systems have greatly advanced our understanding of plant-virus interactions and will continue to do so for many years to come. At the same time, they come with limitations as plant-virus interactions are influenced by the specific virus-host combination, by mixed infections with other pathogens and by environmental factors. Thus, the findings derived from model systems under controlled conditions will need to be re-examined in the context of complex ecosystems, a formidable challenge.

Infecting a plant is not an easy affair. In addition to common tasks shared with viruses infecting other hosts, e.g., learning to borrow, steal or modify host factors to translate, transcribe and replicate their genome, plant viruses face additional hurdles (Palukaitis et al., 2013; Elena et al., 2014). Because plants are not mobile, viruses use insect, arthropods, nematode, or fungal vectors to reach their hosts and have been shown to manipulate the plant physiology and/or the vector behavior to enhance their transmission (Blanc and Michalakis, 2016; Dietzgen et al., 2016). Plant viruses encode specialized movement proteins to modify the narrow plasmodesmata channels that traverse the thick cell wall, thereby allowing cell-to-cell movement of viral RNA complexes or virus-like particles (Harries and Ding, 2011; Heinlein, 2015). Finally, plants deploy an arsenal of antiviral defense responses that viruses must counteract or evade. These include RNA silencing and salicylic acid-mediated responses directed by dominant resistance genes (Carr et al., 2010; de Ronde et al., 2014; Csorba et al., 2015; Faoro and Gozzo, 2015; Gouveia et al., 2016).

Plant viruses normally have a small genome with limited coding capacity (4–20 kb, encoding 5–10 proteins). An explosion of recent studies has highlighted the complexity of these small genomes and the multifunctional characteristics of plant virus proteins. Well-studied examples of multi-functional viral proteins could include the potyvirus HC-Pro protein, the caulimovirus P6 protein, the cucumovirus 2b protein and the tombusvirus p33 protein. HC-Pro is a protease, an aphid transmission factor and a suppressor of RNA silencing (Valli et al., 2017). P6 is a translational trans-activator, a silencing suppressor and a facilitator of cell-to-cell movement (Bonneville et al., 1989; Schoelz et al., 2016). 2b is a silencing suppressor. It also manipulates the jasmonic acid defense pathway and volatile emissions to render plants more attractive to insects (Groen et al., 2016; Wu et al., 2017) and represses the abscisic acid-mediated pathway to enhance drought tolerance (Westwood et al., 2013). p33 regulates various steps of viral RNA replication, acts as an RNA chaperone and remodels intracellular membranes to form replication complexes (Nagy, 2016). To accomplish these functions, viral proteins depend on an extensive network of interactions with other viral proteins and with plant proteins, hormones, lipids, organelles, and intracellular membranes (Laliberté and Sanfaçon, 2010; Wang, 2015; Nagy, 2016). Similarly, viral genomes contain a variety of *cis-*acting sequences or structures that regulate their translation, transcription, replication, and encapsidation via interactions with viral and host proteins (Simon and Miller, 2013; Newburn and White, 2015). The extent of these interaction networks is only beginning to be appreciated. For example, the p33 protein is known to interact with a minimum of a 100 host proteins, with plant lipids and with intracellular membranes (Nagy, 2016).

These interactions must be regulated in a temporal and spatial manner for the virus to complete the sequential steps of its infection cycle. Viral genomes and proteins are highly plastic allowing them to adopt alternative conformations and control their interaction network in response to changes in the microenvironment e.g., increasing accumulation of viral products, induction of plant defense responses or fluctuating environmental conditions (Nicholson and White, 2015; Nagy, 2016). Alternatively or perhaps in addition, a commitment model has been proposed for the p33 protein, in which specific individual molecules interact with different partners allowing multi-tasking of the overall population without having to resolve conflicting interactions on the same region of the protein (Nagy, 2016). As an extension of this model, it would be interesting to examine whether minor variants present in complex virus populations can contribute to expanding the repertoire of interactions between virus components and host factors. Viruses normally exist in highly dynamic quasi-species clouds that contain varying ratios of minor variants (Elena et al., 2014). Although most functional studies have been conducted using infectious clones that initiate virus infection with a uniform genome sequence, the potential contribution of minor variants present in quasi-species clouds on the temporal and spatial regulation of the infection cycle is an area that merits more attention in the future.

Elucidating interaction networks has been rewarding not only because they have deepened our understanding of plant virus infection processes but also because they have opened new avenues for engineering disease resistance. One well-documented example ensued from the identification of an isoform of eIF4E, a plant translation initiation factor, as an interactor of the potyvirus VPg protein (Wittmann et al., 1997). As it turned out, many plant recessive resistance genes were later mapped to eIF4E alleles that are defective in their ability to interact with the VPg but functional for plant mRNAs translation (Truniger and Aranda, 2009). When natural resistance is not available, it can be engineered by down-regulation or targeted mutation of specific EIF4E isoforms (Wang and Krishnaswamy, 2012; Sanfaçon, 2015). Additional translation factors as well as other host factors are emerging as promising new antiviral targets (Wang, 2015; Hashimoto et al., 2016). However, closely related plant proteins are often encoded by multi-gene families and the specificity of interactions can vary with each virus-host combination as exemplified with the VPg-eIF4E interaction (Sanfaçon, 2015). Thus, follow-up studies in relevant crops are required before applications can be developed. The availability of deep-sequencing tools to examine natural variations in plant genes combined with powerful gene editing technologies can inform the design of the next generation of tailored mutations in critical host factors (Bastet et al., 2017).

Given the multiplicity of concurrent and inter-connected plant defense responses, studying the interaction of viruses with these responses has been both challenging and rewarding (Carr et al., 2010; Faoro and Gozzo, 2015; Moon and Park, 2016). RNA silencing, a ubiquitous gene regulation mechanism, is undoubtedly the best studied plant antiviral response. An antiviral role for RNA silencing was first supported by two observations: it is associated with symptom recovery, a natural phenotype of some plant-virus interactions characterized by the emergence of asymptomatic leaves in late infection stages of otherwise diseased plants (Covey et al., 1997; Ratcliff et al., 1997) and it is counteracted by viral silencing suppressors (Anandalakshmi et al., 1998). The characterization of these suppressors has been a fruitful area of research in the last two decades. Most plant viruses encode at least one suppressor although their mode of action varies greatly (Csorba et al., 2015). This is probably a reflection of the diversity of RNA silencing pathways, which are orchestrated by proteins encoded by multigene families. To choose ARGONAUTE proteins as an example, the number of functional *argonaute* genes varies depending on the plant species (10 in *A. thaliana*) and they play distinct, yet sometimes redundant roles in the antiviral response (Mallory and Vaucheret, 2010; Brosseau and Moffett, 2015; Carbonell and Carrington, 2015). Most silencing suppressors were characterized by expressing individual viral proteins in model hosts. Such overexpressed suppressors can cause pleiotropic effects on the plant physiology by interfering with multiple RNA silencing pathways and this has been correlated with symptom expression during natural infections. However, the tombusvirus p19 suppressor was recently shown to impact the plant physiology differently depending on whether it was expressed individually or in the presence of virus infection (Kontra et al., 2016), highlighting the limitations of simplified model systems and reinforcing the need to re-examine the function of viral proteins using more holistic approaches.

RNA silencing should not only be viewed as an antiviral mechanism that must be counteracted. In fact, many viruses encode weak or transiently active suppressors and probably use RNA silencing to limit their accumulation and prevent catastrophic damage to their hosts, as evidenced in the case of symptom recovery (Ghoshal and Sanfaçon, 2015). Virus-derived small RNAs are the by-products of RNA silencing that guide ARGONAUTE proteins to target RNAs and they occasionally share sequence complementarity with plant mRNAs, providing viruses (or viroids) with another tool to fine-tune plant gene expression (Shimura et al., 2011; Navarro et al., 2012; Miozzi et al., 2013; Adkar-Purushothama et al., 2015). Characterizing the impact of the down-regulation of plant mRNAs by viral small RNAs on the outcome of infection is likely to be a focus of attention in the future.

Although relatively well-characterized in model hosts, our understanding of antiviral defense responses is lagging in more complex crops, particularly in trees. The impact of environmental conditions and mixed infections on the outcome of plant-virus interactions is also becoming evident (Palukaitis et al., 2013; Mascia and Gallitelli, 2016). Evaluating these interactions under complex field conditions will be a difficult but necessary endeavor, especially considering changing climatic conditions that will likely be accompanied with increased pathogen pressure (Newton et al., 2012; Jones, 2016).

In this article, I have discussed plant-virus interactions with a focus on pathogens of agricultural crops. However, recent metagenomic studies have revealed the large diversity of viruses found in wild plants, an understudied area of research (Roossinck, 2016). Perhaps not surprisingly, many natural infections do not have detrimental effects on the host. Some are even beneficial (Roossinck, 2015b). These studies have highlighted the delicate equilibrium between plant viruses and their natural hosts that arose from long-term co-evolution and that can be broken by large-scale monocultures. Thus, characterizing the impact of virus infection in natural ecosystems may reveal new beneficial uses of plant viruses and help develop more informed agricultural practices (Roossinck, 2015a).

Mostly using simplified model systems, we have learned much on the intricacy of plant-virus interactions since the discovery of the first (plant) virus. The next phase of plant virology research promises to be exciting as we obtain a better appreciation of the overall impact of viruses not only in model plants, but also in agricultural crops and complex ecosystems.

## Author contributions

The author confirms being the sole contributor of this work and approved it for publication.

### Conflict of interest statement

The author declares that the research was conducted in the absence of any commercial or financial relationships that could be construed as a potential conflict of interest.
